# Molecular Mechanisms and Treatment Strategies of ALK‐Positive Lung Cancer: A Beginner's Guide for Patients, Their Families and Carers

**DOI:** 10.1111/1759-7714.70182

**Published:** 2025-11-10

**Authors:** Elena Klenova

**Affiliations:** ^1^ School of Life Sciences University of Essex Colchester UK; ^2^ ALK Positive Lung Cancer Charity Raglan UK; ^3^ Oncogene Cancer Research Charity London UK

**Keywords:** ALK—Anaplastic lymphoma kinase, EML4—Echinoderm microtubule‐associated protein‐like 4, NSCLC—Non‐small cell lung cancer, RTK—Receptor tyrosine kinase, TKI—Tyrosine kinase inhibitor, TK—Tyrosine kinase

## Abstract

This review has been written with the intention of explaining to the patients with ALK‐positive lung cancer, and to their families, friends, carers and medical teams, in simple terms, the fundamentals, and the current state of knowledge of this particular type of cancer. The review begins with basic facts about lung anatomy and lung cancer, then explains general principles of how cell proliferation is regulated at the molecular level. The coverage of the molecular events underlying the development of ALK‐positive lung cancer and principles of targeted therapies then follows. The review concludes with an analysis of various therapeutic approaches to treat ALK‐positive lung cancer. The Supporting Information section contains additional advanced information illustrating specific points of interest.

## Graphical abstract

**Inhibition of EML‐ALK function by TKI**. In cells, not treated with TKI, ATP binding occurs in the active site (pocket) of the hybrid protein, leading to its activation and functional effects. Introduction of a TKI leads to TKI compete with ATP for binding to the active site. Keys: ALK ‐ Anaplastic Lymphoma Kinase; EML4 ‐ Echinoderm microtubule‐associated protein‐like; ATP ‐ Adenosine triphosphate; TK ‐ Tyrosine Kinase; TKI ‐ Tyrosine Kinase Inhibitor.

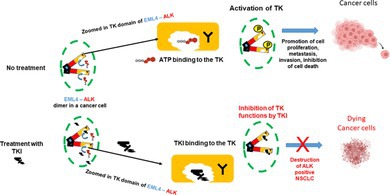

## 1 Introduction

The main aim of this review is to provide readers with the current knowledge and understanding of ALK‐positive lung cancer and its treatment. It is a complex topic and many important concepts are not systematically covered in the literature. The author of this article is an ALK‐positive lung cancer patient with a background in cancer research and education. As a scientist, the author was very curious about the molecular mechanisms of this type of cancer and also keen to explain those to other patients. Existing articles in the professional scientific and medical space often rely on the background knowledge of their readers, and patients (and also many health care professionals) with no special education in these areas do not always find it easy to understand. This review is intended to bridge this gap by explaining in one article, in simple terms, important molecular aspects of ALK‐positive lung cancer and therapeutic strategies for its treatment. The author hopes that this article will be interesting, informative and valuable to patients with ALK‐positive lung cancer, their families, friends, carers, and medical teams. This knowledge may not only help the audience understand the basics of the biology of ALK‐positive lung cancer, but also have an impact on decision‐making and treatment management thereby contributing to improved healthcare outcomes.

The author expects that after reading the review the reader will be able to achieve the following learning goals:

- Be familiar with the review's methodology (Section 2);
- Learn basic facts about lung anatomy and lung cancer (Section 3);
- Learn about general aspects of cancer as a disease (Section 4);
- Learn about basic aspects of ALK‐positive lung cancer (Section 5);
- Understand basic mechanisms of cell response to signals controlling cell proliferation and activation of signaling pathways (Sections 6, 7);
- Learn about specific molecular events taking place in the cell during signal transmission through Receptor Tyrosine Kinases, RTKs (Section 8);
- Learn about ALK function in normal and cancer cells, and about molecular events underlying the development of the ALK‐positive lung cancer (Section 9);
- Understand the concept of Tyrosine Kinase Inhibitors (TKIs) as therapeutic medicines targeting ALK‐positive lung cancer, their development and applications (Section 10);
- Learn about current and future treatment perspectives: future TKIs, surgery, immunotherapy and ALK vaccines, drug combination therapies and novel therapy ideas (Section 11);
- Understand the pending questions, challenges and limitations of our current knowledge of the topic in general and this review in particular (Section 12);
- Learn more about specific points of interest in the Supporting Information S1, (Sections 13–17) containing additional advanced information about ALK protein structure, different variants of *EML4*–*ALK* fusion partners, chemical structures of different ALK TKIs, ALK resistance mutations and treatment algorithms for ALK patients.

## 2 Methods: Information Sources and Search Strategies

This article represents a narrative, not a systematic review. Data, evidence and facts presented here are based on the information obtained from well‐known and respected textbooks [1, 2, 3, 4], websites and original articles. The textbooks provided the material and content to explain lung anatomy and general concepts of cell biology (Sections 3 and 4), control of cell proliferation and mechanisms of signal transmission in normal cells (Sections 6, 8). A systematic literature search was performed in PubMed and Web of Science bibliographic databases. The literature searches covered articles up to August 2025. The search used a combination of the following terms: “lung cancer” and “ALK‐positive,” “Non‐small cell lung cancer” and “ALK‐positive,” “TKI and ALK‐positive and NSCLC,” “ALK inhibitor and ALK‐positive and NSCLC,” “ALK‐positive NSCLC and targeted therapies.” Websites of health organizations and health governing bodies such as NICE (National Institute for Health and Care Excellence) in the UK and FDA (Food and Drug Administration) in the USA were used to obtain specific information about therapies and clinical trials (https://www.nice.org.uk/ and https://www.fda.gov/, respectively). General and specialized educational websites for patients with lung cancer and, in particular, the ALK‐positive subtype were searched to address patients' perspectives and needs. They included Cancer Research UK (https://www.cancerresearchuk.org/), Roy Castle Lung Cancer Foundation (https://roycastle.org/), ALK Positive Inc. (https://alkpositive.org/), ALK Positive Lung Cancer UK Charity (https://www.alkpositive.org.uk/) and Oncogene Research UK Charity (https://www.oncogeneresearch.org/). The original papers were selected according to pertinence; priorities were given to more recent articles containing references to earlier papers.

## 3 Basic Facts About Lung Anatomy and Lung Cancer

**FIGURE 1:**
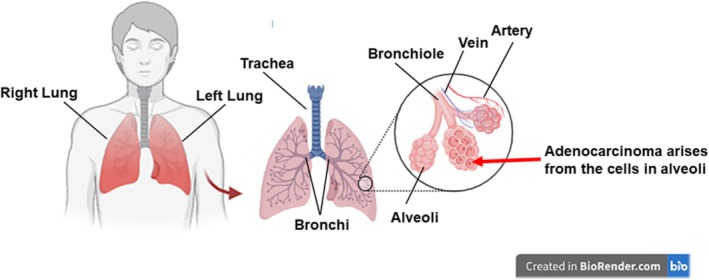
Structure of the respiratory system (based on the information from [4]). (Image is created in BioRender.com).

Lungs are part of the respiratory system, which allows us to breathe [4]. The main role of this system is to bring oxygen to the body and remove carbon dioxide. The general anatomy of the lungs is shown in Figure 1. Air enters the lungs via the trachea (windpipe) then goes into the bronchi, ending in small air sacs (alveoli). The alveoli are made of several types of cells, which are responsible for the transfer of oxygen from the air into the bloodstream. Adenocarcinoma, which belongs to the category of non‐small cell lung cancer (NSCLC), arises from epithelial cells secreting mucus that are lining the alveoli.

## 4 Basic Facts About Cancer

**FIGURE 2:**
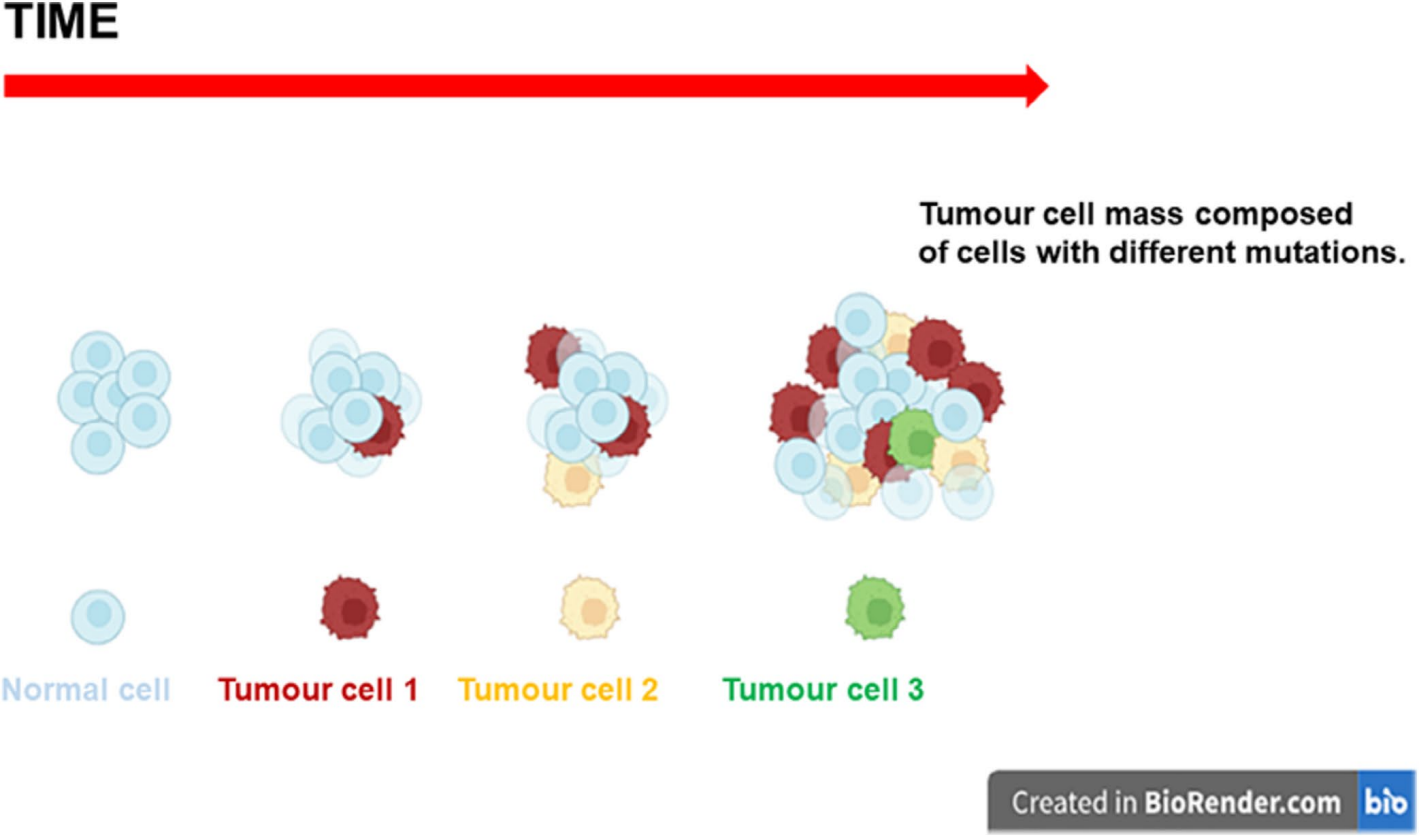
Multistage evolution of cancer. See the text for explanations. (Image is created in BioRender.com).

The fundamental feature of cancer is the continuous increased proliferation (division) of cancer cells. It results from cancer cells losing their ability to respond appropriately to the signals controlling the behavior of normal cells. The development of cancer is a multistep process illustrated in Figure 2. It starts with one cell acquiring an initial mutation giving this cell the ability to multiply continuously. A high rate of proliferation, which in turn increases the risk of mutations, may result in the acquisition of the second mutation in some of the descendants. Cells with the double mutation may have selective advantages (i.e., they can proliferate faster). Additional mutations can lead to further evolution of cancer; for example, cells may gain the ability to spread (metastasize). The resulting cancer cell population is heterogeneous, i.e., it consists of cells carrying different properties. Due to such variations, it is very challenging to treat cancer since some cells may not respond to the therapies.

## 5 General Facts About ALK‐Positive Lung Cancer

ALK (Anaplastic Lymphoma Kinase)‐positive NSCLC is a unique molecular subtype of lung cancer involving the rearrangement of the *ALK* gene (see later sections of the review for detail). It is estimated to contribute to about 4% of all NSCLC [5], however, the ALK‐positive NSCLC incidences are not uniform and differ geographically ranging widely between 0% and 19.6% [6]. Patients tend to be younger (median age 52 years), female, never or light smokers and histologically adenocarcinoma dominated. Most ALK‐positive patients are diagnosed with advanced disease, with the high incidence of metastases to the brain, which may reflect the aggressiveness of these tumors ([7, 8] and references therein).

## 6 Signals Controlling Cell Proliferation

**FIGURE 3:**
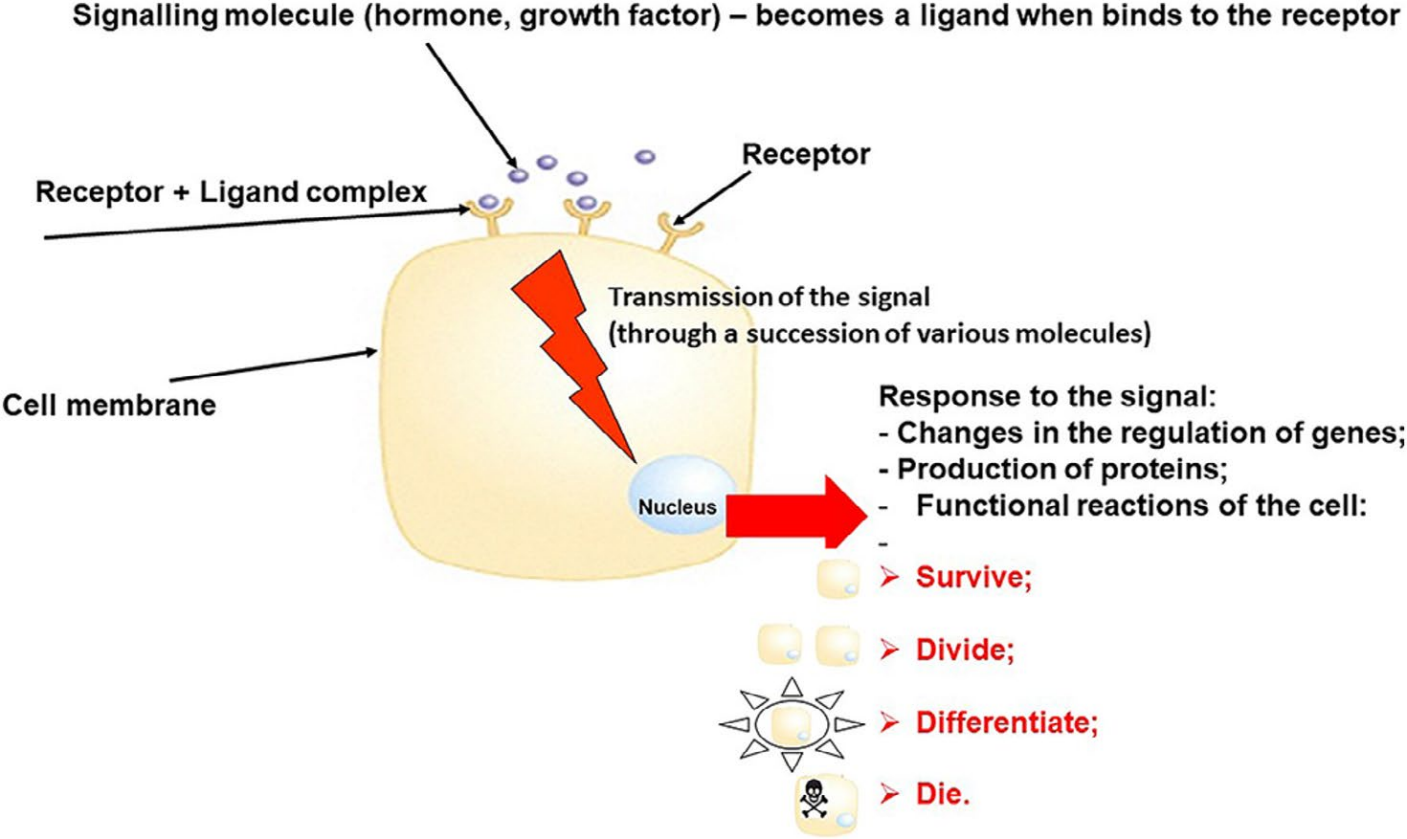
Cell response to regulatory signals (steps include receptor‐ligand interaction, signal transmission and cell response). (See the text for details).

In multi‐cellular organisms, decisions about an individual cell's growth and no‐growth are made “in consultation” with other cells in the tissue. To communicate their messages, the latter release growth regulatory signals (hormones, growth factors etc.). Normal cells receive these signals through specific molecules on their surface, called receptors. Once bound to a receptor, the signaling molecule becomes known as the ligand. In the cells, signals are transmitted, processed and integrated by a succession of signaling molecules, which are organized in signaling pathways. As a result, a specific biological response will be achieved, regulating survival, division, differentiation (“maturation”) or death of the cell. Deregulation of one or more of the steps in these pathways can lead to uncontrolled cell division and the formation of a tumor. These processes are illustrated in Figure 3.

## 7 Activation of the Signaling Pathways: Role of Kinases (With the Focus on Tyrosine Kinases)

**FIGURE 4:**
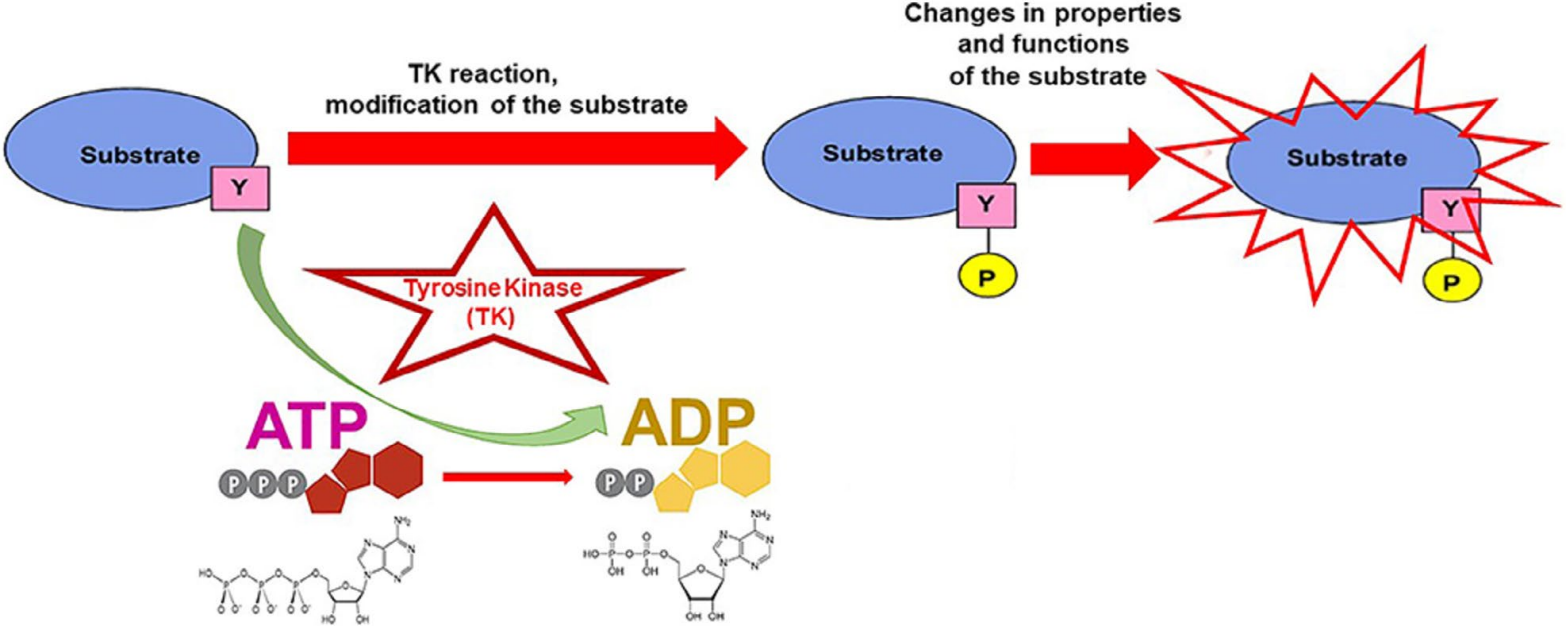
Modification of the substrate with Tyrosine Kinase (TK). The TK interacts with the substrate and then breaks the ATP molecule to transfer one of the phosphate groups to the amino acid Tyrosine within a protein molecule. As a result of this reaction, Tyrosine is modified, or phosphorylated; this modification changes the properties of the whole substrate molecule. Keys: Substrate, the substance on which the kinase acts; Y, amino acid Tyrosine; P, phosphate group added to Tyrosine; ATP, an energy‐rich molecule with three phosphate groups; it is the donor for the phosphate group. (The other two groups are a nitrogenous base (adenine) and a ribose sugar). ADP, a molecule with two phosphate groups after ATP is broken. The graphic illustrations of ATP and ADP, together with their structural diagrams, are shown.

A major role in the activation of the signaling pathway belongs to a receptor. It is usually a complex molecule, composed of several functional units (or domains). In this review, we will focus on a class of cell surface receptors that contain, among others, a kinase domain. What is a kinase? A kinase is a type of protein that transfers phosphate groups from high‐energy donor molecules, such as ATP (Adenosine triphosphate) to specific target molecules (substrates); this process is termed phosphorylation. A Tyrosine Kinase (TK) is an enzyme that can transfer a phosphate group from ATP to the Tyrosine residue of specific proteins (Tyrosine is one of 20 amino acids that combine to build proteins). This modification functions as an “on” or “off” switch in many cellular processes. In this reaction, Adenosine diphosphate (ADP), a molecule with two phosphate groups is also produced, after a phosphate group is released from ATP. The process of phosphorylation by TK is illustrated in Figure 4.

## 8 Important Molecular Events Taking Place During Signal Transmission Through Receptor Tyrosine Kinases

**FIGURE 5:**
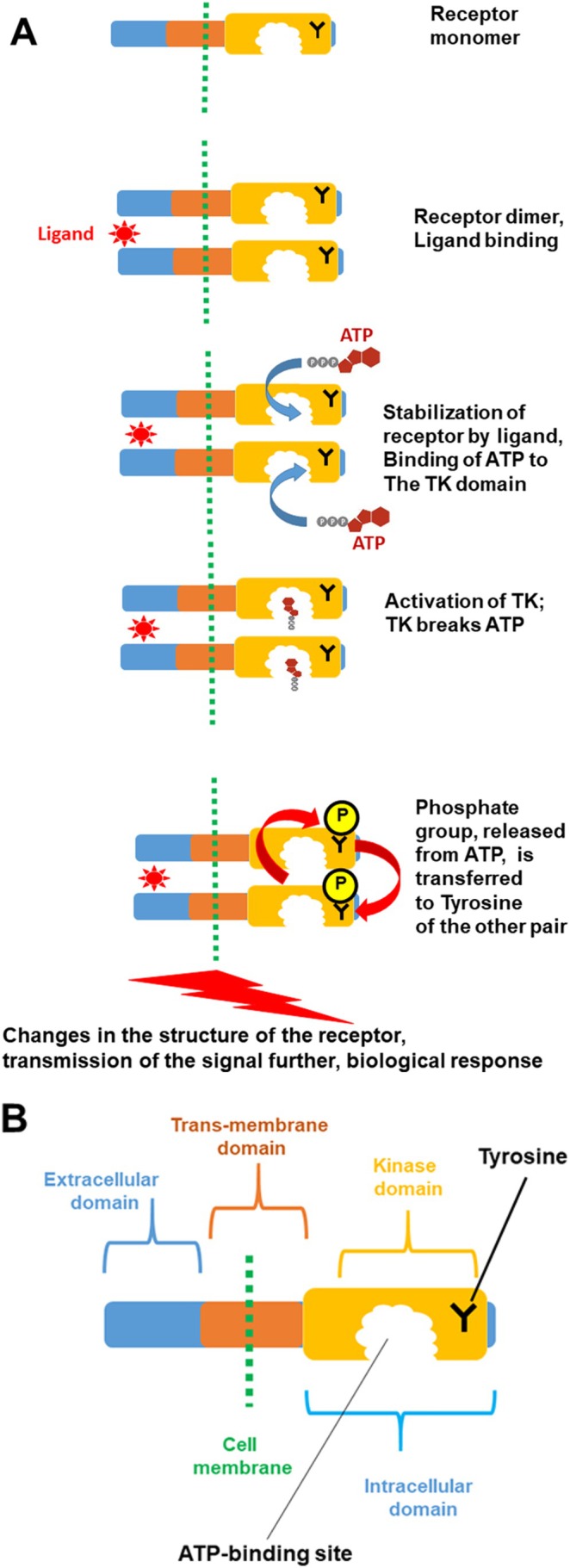
RTK/TK signaling activation. (A) Activation of RTK/TK signaling following the ligand binding. (See the text for detail). (B) Structure of the RTK monomer: Key features are shown.

As discussed in the previous section, receptors are composed of several functional units (domains). As illustrated in Figure 5A,B, a typical receptor, located at the surface of a cell, contains three domains: extracellular (projected outside of the cell and responsible for binding a ligand), trans‐membrane (anchoring the receptor molecule at the membrane) and kinase domain (modifying and activating the receptor). If Tyrosine is modified by the kinase domain, the latter is classified as Tyrosine Kinase (TK) domain. In the scientific literature, a receptor containing the Tyrosine Kinase domain is referred to as Receptor Tyrosine Kinase (or RTK).

In an unstimulated cell, single molecules (monomers) of the RTKs can move freely in the cell membrane. From time to time they encounter another single molecule and form temporary association (dimer). It is usually not stable and can dissociate easily. However, if a cell receives a signal, resulting in a binding of a ligand to the RTK, the bond between the monomers strengthen and the dimer becomes stable. The processes, which follow the dimer formation are described in Figure 5A. The ligand‐dimer RTK complex can now bind ATP molecules; this takes place within a pocket in the TK domain. This binding is very specific, so that ATP fits very accurately into the cavity of the pocket. As a result, TK function is activated; this leads to the release of the phosphate group from ATP, its transfer to Tyrosine of the partner RTK and release of ADP (the details of the kinase reaction are described in Figure 4). The modified RTK dimer changes its spatial structure (conformation) and it is now able to trigger subsequent events leading to changes in gene function, production of particular proteins and, finally, adequate biological response of the cell to the initial signal. In cancer cells the regulatory mechanisms of signal transmission, which are very tight in normal cells, are disturbed leading to increased uncontrolled cell proliferation. Such mechanisms are multiple and complex and discussing them is beyond the scope of the current article. However, some aspects of these processes are described later in relation to the development of ALK‐ positive NSCLC.

## 9 The Function of ALK in Normal and Cancer Cells

### 9.1 The Function of ALK in Normal Human Cells and Tissues

The *Anaplastic Lymphoma Kinase* (*ALK*) gene was originally identified in 1994 and implicated in the development of a particular type of cancer, anaplastic large cell lymphoma [9]. Later, the rearrangements in the *ALK* gene, discovered in a subgroup of NSCLC and called ALK‐positive [10], were demonstrated to be responsible for the development of lung cancer [11]. ALK belongs to the RTK family, and is composed of three domains (extra‐cellular, trans‐membrane and kinase) (Figure 5B) ([12, 13, 14] and references therein). A detailed structure of ALK is shown in Figure S1.

**FIGURE 6:**
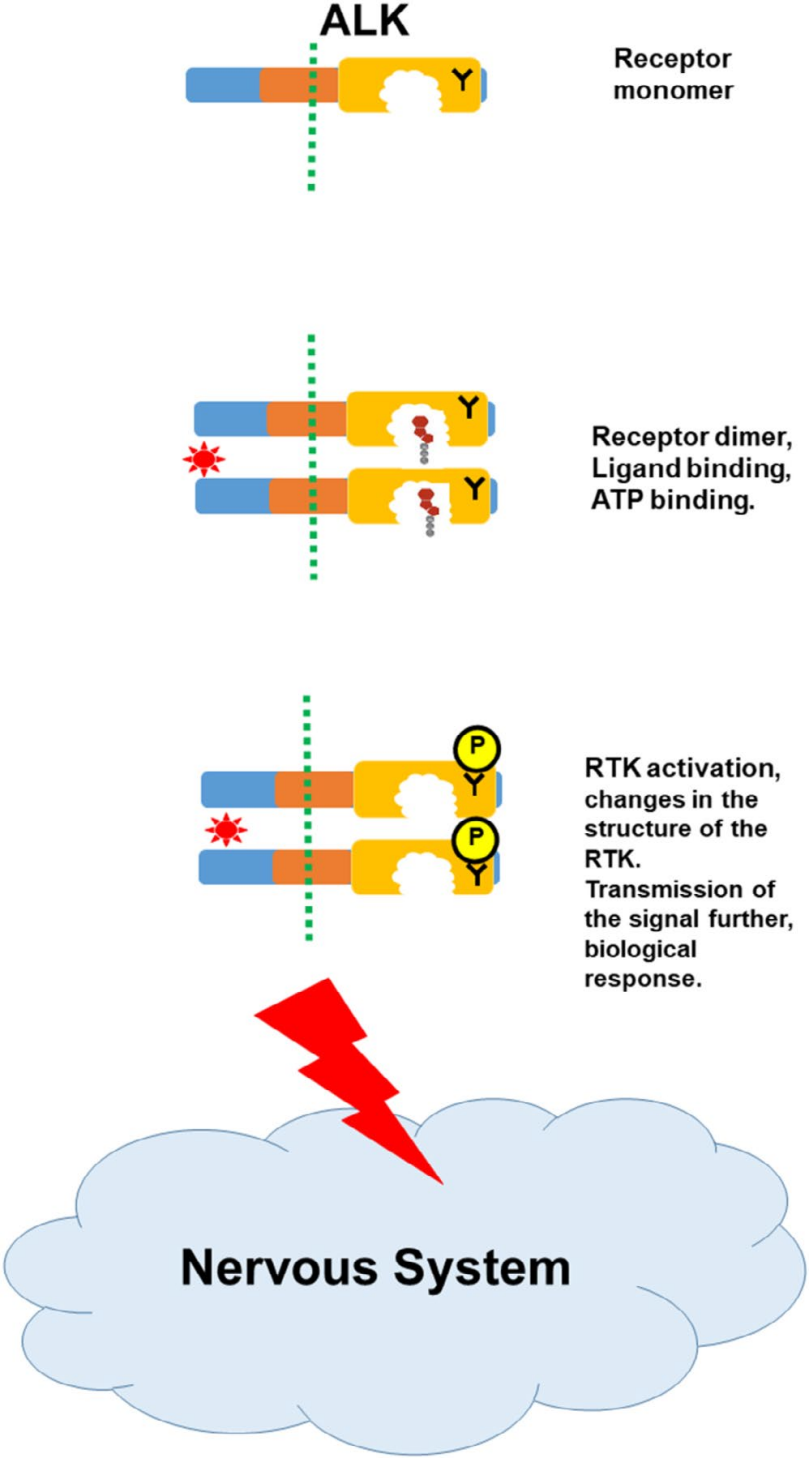
ALK signaling during normal embryonic development (a few key steps are shown). Specific ligands activate ALK leading to regulation of biological functions important for the growth and development of the nervous system [13]. (Key features are as in Figure 5).

ALK is not normally detected in human cells and tissues, except in embryos where it controls the development of the nervous system (Figure 6). There, ALK is regulated by specific ligands, and, typically for the RTKs, two ALK monomers bind to form the ALK dimer and the subsequent events follow as described in detail in a previous section and Figure 5.

### 9.2 ALK in Cancer Cells: Molecular Events Underlying the Development of the ALK‐Positive Lung Cancer

**FIGURE 7:**
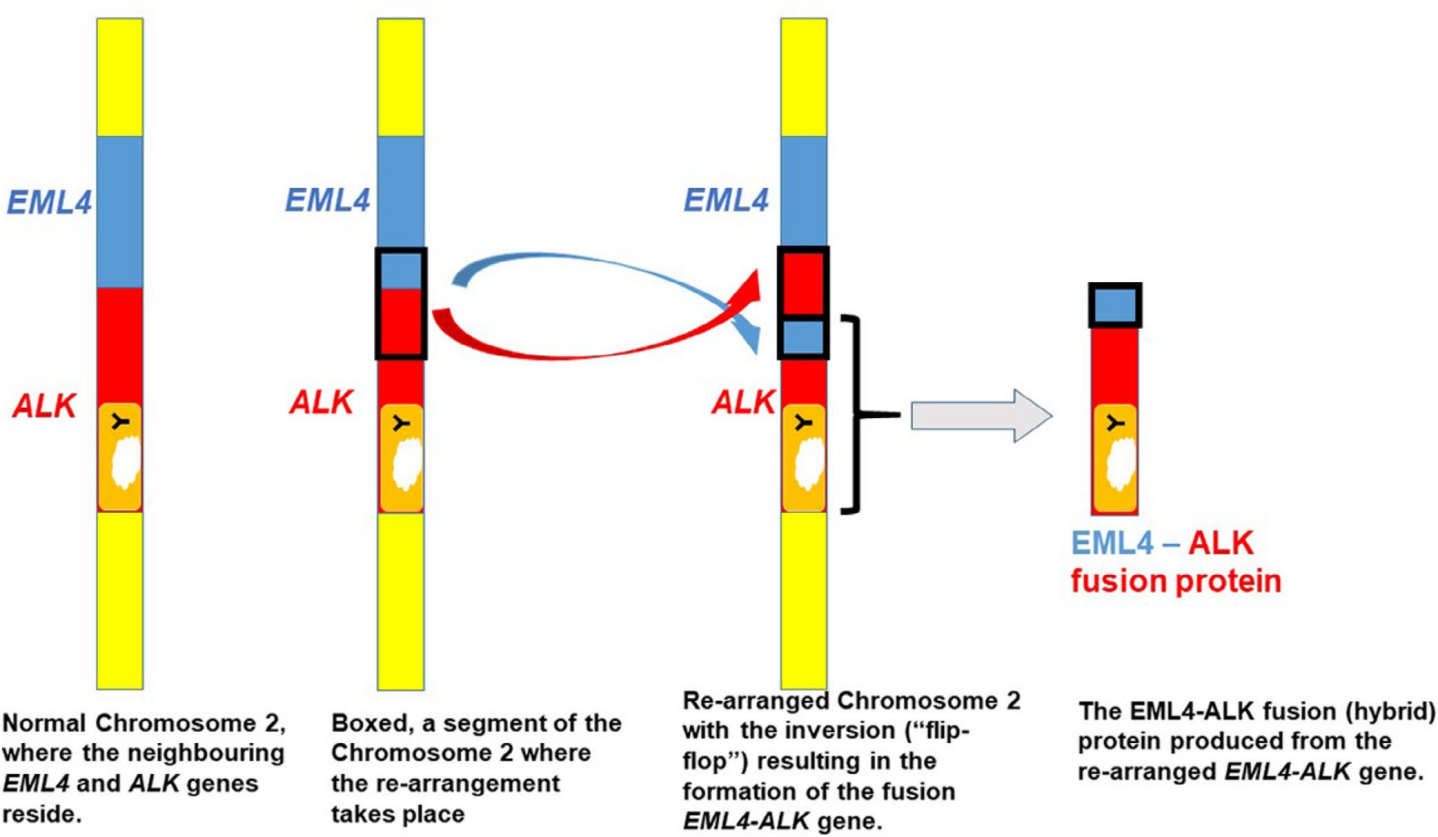
Generation of the *EML4–ALK* hybrid gene due to an inversion within chromosome 2. The segment with the kinase domain from *ALK* is now fused to a part of *EML4*, resulting in the generation of the EML4‐ALK fusion protein. Keys: Blue, EML4; Red, ALK; Orange, the kinase domain of ALK; Yellow, parts of Chromosome 2.

In ALK‐positive lung cancer cells an abnormal configuration in the DNA was discovered, which resulted in the aberrant production of ALK in these cells [15]. Molecular analysis of the DNA from these cells showed that the *ALK* gene is fused to another gene, called echinoderm microtubule‐associated protein‐like 4 (*EML4*). The essence of this change is the inversion, or “flip‐flop” of a DNA segment between these two neighboring genes within chromosome 2 (Figure 7, left panel). A very interesting question is why the breaks take place only in some specific regions. A computational approach to scrutinize places where the rearrangements occur revealed that those sites are particularly “fragile” (i.e., easy to break); hence susceptible to reorganizations [16]. This abnormal gene fusion results in the production of a fusion protein (EML4–ALK) that is the main driver of the malignant behavior [17] (Figure 7, far right).

**FIGURE 8:**
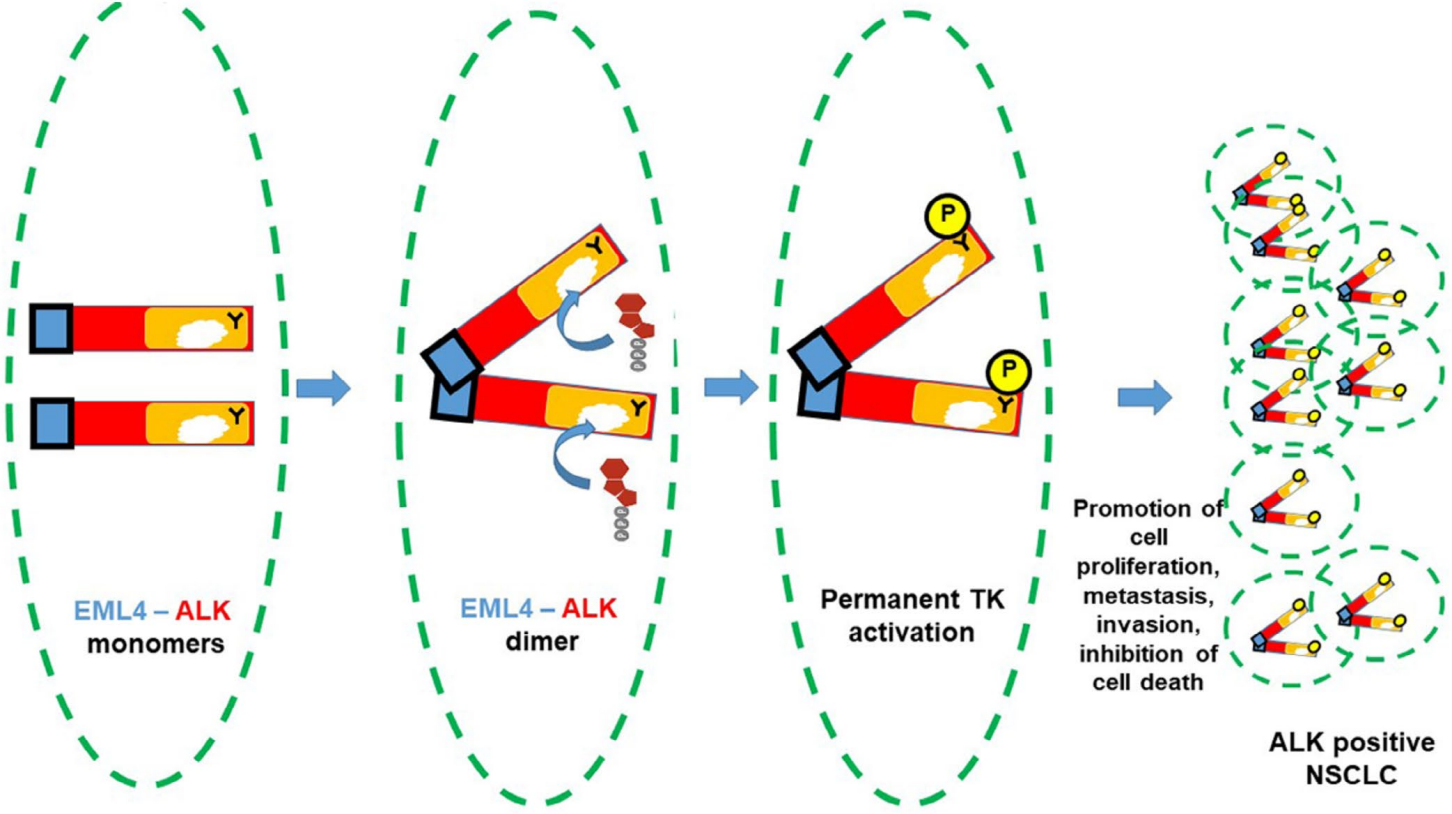
ALK signaling in cells producing EML4‐ALK hybrid protein. The EML4‐ALK protein does not have the extra‐cellular and trans‐membrane domains and is located inside the cells. It does not require a ligand, it can form dimers using the EML4 surfaces, recruit ATP and be directly activated thereby triggering pathways supporting NSCLC development. Keys: Blue, EML4; Red, ALK; Orange, the kinase domain from ALK; Y, amino acid Tyrosine; P, phosphate group added to Tyrosine. Green dashed line depicts the cell membrane.

The EML4–ALK fusion protein does not contain extracellular and transmembrane regions of ALK. This means the EML4–ALK protein is localized to the cytoplasm rather than the cell membrane. It always contains the kinase domain of ALK, but the size of the fragment derived from the *EML4* gene can differ, depending on the breakage point in *EML4*. Around 100 *ALK* fusions with various partners have been reported in ALK‐positive NSCLC, and *EML4–ALK* (with multiple fusion breakpoints in *EML4*) accounts for approximately 95% of *ALK* fusion variants ([18, 19, 20] and references therein). Different variants of *EML4–ALK* fusions and their frequencies are shown in the diagrams in Figure S2. The fusion genes encode a hybrid protein, EML4–ALK, which can form dimers even without the presence of the extracellular ligand [15, 21, 22]. This is due to the fact that the protein regions derived from EML4 carry the features that facilitate interaction between proteins. The phosphorylation of the EML4–ALK follows, leading to the activation of signaling pathways promoting cell proliferation, metastasis and inhibiting cell death. These events result in the occurrence of the ALK‐positive NSCLC (Figure 8).

## 10 Development of Tyrosine Kinase Inhibitors (TKIs) as Therapeutic Medicines Targeting the ALK‐Positive NSCLC

**FIGURE 9:**
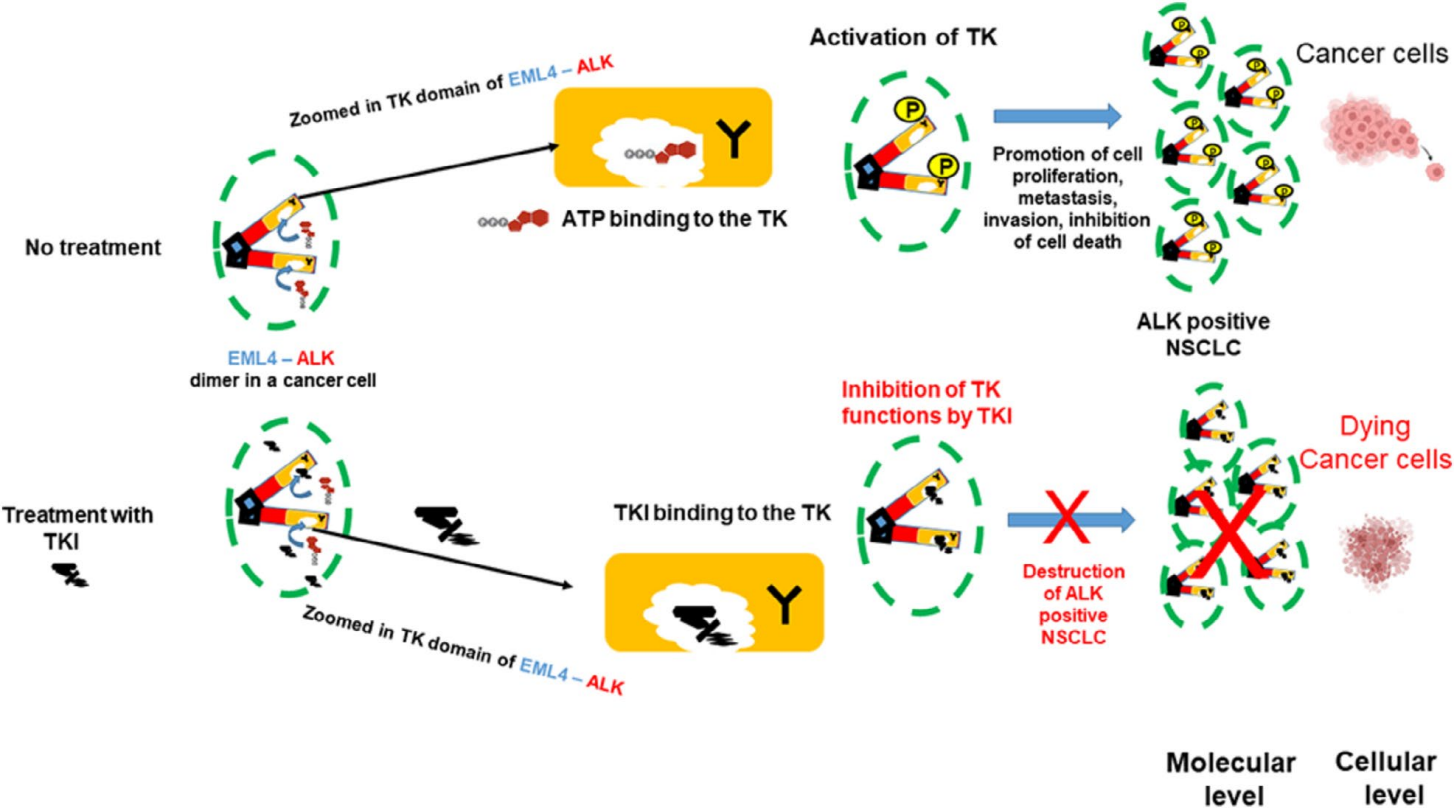
Inhibition of TK function in EML–ALK hybrid protein by TKI. In cells, not treated with TKI, ATP binding occurs in the active site (pocket) of the TK, leading to TK activation and functional effects. Introduction of a TKI leads to TKI competing with ATP for binding to the TK active site (pocket), resulting in the inhibition of the EML–ALK function and destruction of the cancer cells. Keys are as in Figure 8. (Image is created with BioRender.com).

Understanding of the molecular event taking place during the development of the ALK‐positive NSCLC, and, in particular, identification of the EML4–ALK protein as a driver of this type of cancer, paved the way to develop targeted therapies. The rationale behind these is to create molecules (inhibitors) that can block TK function in the fusion EML4–ALK protein. This will lead to its inactivation, inhibition of signaling processes, block of proliferation and even death of cancer cells. In addition, since EML4–ALK protein is only present in cancer cells and absent in normal cells, the TK inhibitors (TKIs) will be highly selective for cancer cells, thereby minimizing side effects of the treatment. The TKIs resemble the ATP molecule and their role is to “trick” the TK by outcompeting ATP and preferentially binding to the TK. The small size of the TKIs is also an important factor as it helps these molecules to pass easily through the cell membrane. Structures of several TKIs used for treatment of ALK‐positive NSCLC are shown in Figure S3; it is notable that their shapes and sizes are comparable to ATP. The TK, once TKI is bound, will not be able to process the signal since the TKI does not have correct functional groups, and hence TK will remain in the “frozen” state. As the activity of the TK is blocked, further events will not take place and there will be no functional response to the signal. This is important because cells will stop proliferating, they may even enter the death pathway and be eliminated. These processes are illustrated in Figure 9.

**FIGURE 10:**
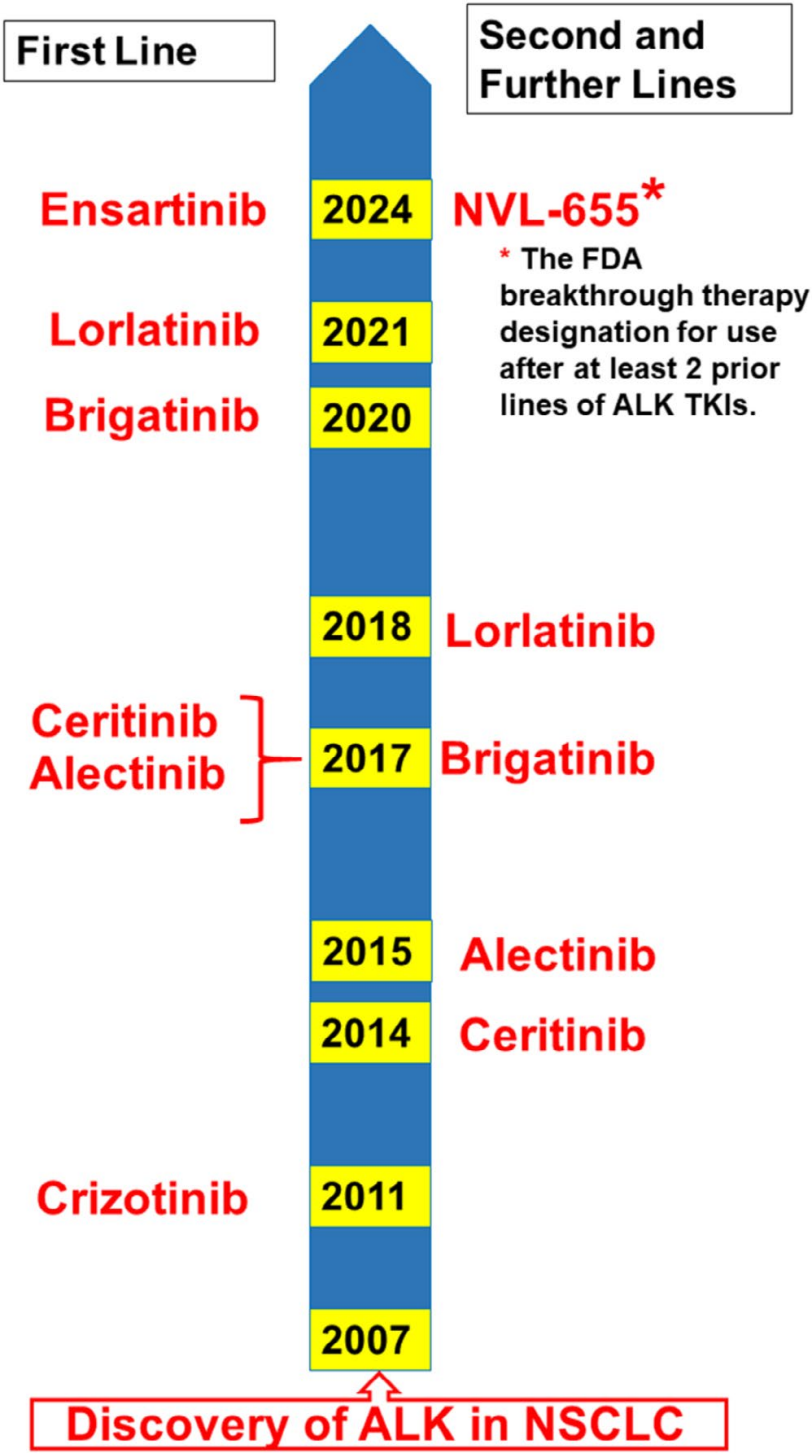
Timeline of US FDA approval of ALK‐positive NSCLC TKIs, based on successful clinical trials. Years of the approval for the first line of treatment by particular TKIs are shown on the left, and second/further lines on the right. Treatment lines refer to the sequence of different TKIs used as cancer progresses. The Food and Drug Administration (FDA), is the United States' regulatory agency for food, medical, and an array of other products.

Crizotinib, the “first generation” (1G) TKI used for the treatment of ALK‐positive NSCLC, was approved by the US FDA in 2011 [23] and by the UK NICE in 2014 [24]. The development and approval of the second (Ceritinib, Alectinib, Brigatinib) and third (Lorlatinib) generations (2G and 3G, respectively) of TKIs followed. Ensartinib, belonging to the second generation, was approved in China in 2022 [25] and by the US FDA in 2024 [26]. The timeline of US FDA approval of different TKIs for their use as first and further lines of treatment is presented in Figure 10 ([27] and references therein).

These medications are highly effective targeted therapies; however, cancer cells evolve by acquiring new mutations and developing resistance leading to disease progression. A growing body of genetic data is being generated by ongoing research to understand molecular changes taking place during treatment with TKIs. Whereas in the initial EML4–ALK fusion protein the ALK TK domain is the same as in the original, the whole ALK protein (also called “wild‐type”), during treatment with TKIs single mutations accumulate in this TK domain. These mutations can disrupt the binding of the TKI to the ALK protein, which can result in treatment failure. Furthermore, after the second and third TKIs the compound (“double”) mutations can occur [23, 28]. A typical profile of the distribution of ALK mutations in samples from patients who developed resistance to different TKIs, as identified by genetic testing, is shown in Figure S4. Interestingly, some mutations are more common than others.

In addition to ALK mutations (“on‐target”), other mechanisms, including the activation of bypass signaling pathways (“off‐target”), can cause resistance. Such a complex mutational landscape creates a substantial challenge for identifying optimal treatment strategies. These aspects will be briefly discussed in the next section.

## 11 Current and Future Treatment Perspectives

### 11.1 Future Tyrosine Kynase Inhibitors (TKIs)

The understanding of the mechanisms of resistance to TKIs is important to design future therapies, and the accumulated knowledge has already revolutionized the treatment of ALK‐positive NSCLC with TKIs ([23, 28, 29] and references therein). The debates among clinicians, regarding the best first‐line TKI treatment of *ALK*‐positive NSCLC are still ongoing. They have recently been intensified following the publication of the results of the CROWN study reporting that 60% of patients receiving Lorlatinib remained on treatment after 5 years [30]. Nonetheless, there is still a strong rationale to identify new TKIs, and many new compounds with the desired properties have been reported in the literature [31, 32, 33, 34]. Phase III ALKAZAR clinical trials of the fourth generation (4G) “double mutant active” TKI, Neladalkib, or NVL‐655 [35], are currently taking place and this TKI is anticipated to be approved (also see Figure 10). The clinical development of another 4G TKI, Zotizalkib, or TPX‐0131, has been withdrawn due to safety issues [36, 37]. A growing number of TKIs have been undergoing clinical trials in China, with more and more of them obtaining approval to use in clinical practice in that country [38]. Regardless of the sequence of current TKI applications, resistance to them will eventually develop due to mutations, single and/or compound, in the *ALK* gene (“on‐target”), and/or in bypass pathways (“off‐target”) [23, 39].

Importantly, different genomic *ALK* variants and mutations demonstrate different sensitivity to therapies ([40, 41] and references therein), therefore in the future it will become common practice to obtain molecular profiles of the ALK‐positive NSCLC from patients throughout their treatment pathways to identify the best treatment options. New technologies, such as molecular analysis of liquid biopsies will therefore become gold standards in clinical practice [42] and provide comprehensive information about specific “on‐target” and “off‐target” mechanisms that cause resistance. One of the proposed treatment algorithms for ALK‐positive NSCLC is presented in Figure S5 [23]. More personalized treatment approaches are currently being discussed in the literature taking into account various risk factors based on factors specific to a patient [43].

A very encouraging example of the clinical value of TKIs is the treatment of one of the blood cancers, chronic myeloid leukemia (CML). The Philadelphia (PH) chromosome, a genetic alteration generating the fusion protein, BCR–ABL1, characterizes CML. BCR–ABL1 is a constitutively activated TK responsible for the pathogenesis of CML. Approved in 2001, Imatinib (or Gleevec), representing the prototype TKI of targeted therapy, has had spectacular success in increasing the life expectancy of CML patients, which now approaches the life expectancy of the general population [44]. Until recently, the therapy of CML with TKIs was not considered curative; however, various recent trial results demonstrated that a proportion of patients can stop treatment with TKI without experiencing a disease relapse and can potentially be cured [45, 46]. It should be noted, however, that the direct comparison of CML (blood cancer) and ALK‐positive NSCLC (solid tumor) is not accurate in some aspects. Nevertheless, the knowledge gained by scientists and clinicians over more than two decades of TKIs application for CML treatment will hopefully guide and facilitate progress in the targeted treatment of ALK‐positive NSCLC.

### 11.2 Surgical Resection of Primary Lung Cancer in Patients With Residual Disease After Previous Systemic Treatment

Local consolidative therapy (LCT), surgery or radiotherapy, for patients with stage IV NSCLC and limited metastatic disease burden has been used successfully in clinical practice [47, 48]. Could surgery to remove the original (primary) tumor site be an option for ALK‐positive NSCLC patients treated with TKIs? The rationale for this is that despite the efficacy of TKIs, there is usually persistence of minimal residual disease and acquired drug resistance inevitably occurs [5]. The molecular basis of the therapy‐induced residual disease is not well understood; the surviving dormant cell population has been shown to include non‐ or slow‐proliferative cells (e.g., cancer stem cells, senescent and quiescent differentiated cancer cells, among others) that are not sensitive to division‐inhibiting therapies [49, 50]. These cells are thought to contribute to therapy resistance and tumor recurrence, and therefore their eradication is paramount. Under current treatment guidance, chemotherapy is usually offered after all TKIs are exhausted. Recent reports demonstrated that surgical resection of primary lung cancer performed before resistance to TKIs develops can improve clinical benefits for these patients [51]. In another paper, an ALK‐positive NSCLC patient with multiple organ metastases who had chemotherapy and targeted therapy, was successfully treated with surgical resection and obtained treatment‐free remission (TFR) for more than 3 years [52]. In these studies the NSCLCs were down‐staged following chemo‐ and/or targeted therapies, so that active cancer cells were detected only in the primary site. Recently, a case was reported, when surgery was performed after a patient had progression on Alectinib. Lorlatinib was used as the second‐line treatment after surgery, but it was discontinued due to toxicity. However, the patient still had no lung cancer recurrence 14 months after discontinuation [53]. Another case describing an example of surgery performed in the UK for a patient on TKI for initially unresectable ALK‐positive NSCLC was recently reported [54]. An extended study, the clinical trial BRIGHTSTAR, involving larger groups of patients diagnosed with oncogene‐driven stage IV ALK‐positive NSCLC and treated with Brigatinib, reported benefits of surgical resection of the primary tumor [55]. These data show promise in using surgery for treating ALK‐positive NSCLC. Significantly, the Society of Thoracic Surgeons (STS) has recently published a clinical practice guideline on the management of oligometastatic NSCLC, emphasizing patient selection and the need for coordinated individualized care [56]. This document supported similar recommendations from other organizations (namely, ESMO‐MCBS and ASTRO/ESTRO) [57, 58, 59].

### 11.3 Immunotherapy and ALK Vaccines

The role of the immune system in cancer development and treatment has been increasingly recognized. Various immunotherapies assisting the immune system in fighting cancer cells have been designed and successfully employed. Immunotherapy with monoclonal antibodies (mAbs) can block programmed death 1 and programmed death ligand 1 (PD‐1/PD‐L1)–dependent negative regulation of the immune response and has been important in the treatment of particular types of lung cancer. In pre‐clinical studies, the presence of EML4–ALK was found to be associated with increased levels of PD‐L1 [60] and, therefore, treatment of ALK‐rearranged NSCLC with immune checkpoint inhibitors (ICI) was expected to be an effective therapeutic strategy. In clinical studies, however, levels of PD‐L1 in ALK‐positive NSCLC tissue samples varied broadly and treatment with ICI was inefficient in ALK‐positive NSCLC [61]. In fact, the poorest response to ICI was observed in patients with the highest levels of PD‐L1. The reason for this phenomenon is likely to lie in the immunosuppressive tumor microenvironment in ALK [62, 63, 64]. Research and pre‐clinical studies are currently ongoing to understand mechanisms by which ALK‐positive NSCLC escapes host immunity and therefore do not respond to immunotherapies [62, 64].

The chimeric antigen receptor (CAR)–T cell therapy, which has been effective in the treatment of hematologic malignancies, encountered significant challenges when applied to solid tumors, including ALK‐positive NSCLC. Factors such as the necessity for ALK‐positive NSCLC to carry “potent” tumor‐specific proteins to provide a response to CAR‐T cells, the complexities of the tumor microenvironment, obtaining an appropriate CAR structure together with the difficulties for CAR‐T cells to enter a solid tumor, contributed to the significant delay in the development of these immunotherapeutics [62].

The concept of ALK vaccines for the treatment of ALK‐positive NSCLC is to trigger a specific immune response against ALK‐fusion proteins [65, 66]. Since ALK is not normally present in human cells and tissues (except in embryos), but appears in cancer cells, it makes it a very promising tumor‐specific target for immunotherapy in ALK fusion‐positive NSCLC. In pre‐clinical studies using mouse models, an ALK vaccine was shown to be very effective [67]; this knowledge is currently being used to apply similar methodology to the human system, with the plan to begin clinical trials in the near future. A phase I clinical trial is currently ongoing to test another ALK vaccine for ALK‐positive NSCLC [68].

### 11.4 Drug Combination Therapies

It is important to acknowledge that the quest to discover novel therapeutic strategies to treat ALK‐positive NSCLC is ongoing. One of them is to develop combinatorial approaches, whereby ALK TKI is combined with another drug attacking the secondary target to enhance the treatment efficacy [69, 70]. Combination of ALK TKI with immune checkpoint inhibitors (ICI) demonstrated encouraging preliminary results, although the issue of toxicity remains to be resolved [23, 71]. Another approach is to use ALK TKI together with inhibitors of angiogenesis that prevent tumor growth by blocking the signals responsible for the development of blood vessels supplying the tumor with oxygen. This strategy provided excellent outcomes when applied to patients with EGFR‐positive NSCLC. Several ongoing clinical trials are being carried out to determine the efficacy of this combination in the treatment of ALK‐positive NSCLC [72]. Chemotherapy with ALK TKI is yet another example of a combined treatment; the phase II trial, B‐DASH, to evaluate the efficacy of Carboplatin, Pemetrexed and Brigatinib in ALK‐positive NSCLC is ongoing in Japan [23, 73]. Combining ALK TKIs with radiotherapy is also being explored and these studies show promise [70, 74]. Finally, ALK TKI in combination with drugs targeting other members of activated ALK‐dependent and also ALK‐independent (bypass) signaling pathways is an exciting treatment avenue that attracts attention from scientists, clinicians and the pharmaceutical industry [70]. Among emerging new therapeutics dual‐target inhibitors (a single molecule that can target two distinct pathways) show promise; they may reduce systemic toxicity and mitigate resistance mechanisms in cancer treatment [75].

### 11.5 Novel Therapy Ideas

In addition to existing treatments, a number of promising novel therapeutic agents are in the pipeline and more are coming from research and innovation [39, 76]. Examples of such approaches currently in the experimental phase are Proteolysis Targeting Chimeric Molecules (PROTACs), antibody drug conjugates (ADCs) [35], immune modulating strategies, oncolytic virus therapy [77], and activation of anti‐tumor immune response through administration of a generalized RNA vaccine [78]. In this context, detection of the molecular changes occurring in ALK‐positive NSCLC will become imperative to design the most appropriate, patient‐tailored treatment options.

## 12 Discussion

### 12.1 Socio‐Economic, Health and Research Challenges

Despite the significant progress made to date to combat ALK‐positive NSCLC there are still many pending questions and challenges that patients, scientists and clinicians face. An important socio‐economic aspect of ALK‐positive NSCLC is that it affects young people in the prime of their lives, at the height of careers and with dependent families. These patients have to combine and balance treatment with work and family responsibilities. Having a better understanding of their condition and available treatments can help overcome the feeling of uncertainty and bring more confidence for the future. The development of resistance to TKIs is a serious challenge, but it is encouraging that several TKIs are available to patients and the development of the new TKIs is in progress. Another challenge in treating ALK‐positive NSCLC is the persistence of the residual disease at the primary tumor sites in the lung. A growing body of evidence demonstrate that application of surgery and radiotherapy to treat these areas, in combination with the systemic targeted therapy, lead to significant improvement of overall survival and disease control in patients with stage IV ALK‐positive NSCLC [55, 79]. The cause(s) for inefficiency of immunotherapy‐based approaches to treat ALK‐positive NSCLC represent an important pending question. Research in this area is focused to understand why this cancer subtype is “immune‐cold” and how to overcome this resistance [64, 80]. Very little is known what factors, external and internal, can be responsible for the *ALK* gene rearrangements. Environmental factors were reported to be associated with the EGFR‐driven lung cancer, whereby the levels of an air pollutant, particulate matter 2.5 (PM2.5), were associated with higher incidences of this cancer subtype [81]. Similar to the ALK‐positive subtype, the EGFR‐driven lung cancer is also common for never smokers or light smokers. It remains to be established if there is an association with air pollutants and ALK‐positive NSCLC, and how those environmental factors can effect processes at the cellular and molecular levels to cause cancer. It will be important that research efforts continue to understand these pending questions and challenges.

### 12.2 Limitations of This Review

The discovery of the rare ALK‐positive NSCLC subtype is relatively recent; hence many scientific and clinical aspects related to its understanding are still being investigated and the outcomes from ongoing clinical trials are yet to be published. This review is narrative, non‐systematic, reflecting the current situation in the field as still being a work in progress with no definite answers to many questions. It is aimed at a wider audience including patients, their families, friends, carers and medical teams, and therefore is written in simpler language. The review addresses the core principles rather than exploring the specific details of the processes (that could be of more interest to specialists in the field). However, the readers can learn more about particular aspects of ALK‐positive NSCLC from the Supporting Information sections containing additional advanced information and acquire even deeper knowledge from the original sources cited within the paper. The selection of the articles for this review was based on the author's personal background and experiences, which could inadvertently influence the narrative; however the author intended and tried to be as objective and unbiased as possible.

### 12.3 Conclusions

The ALK‐positive NSCLC represents a unique cancer type with very distinct molecular and clinical‐pathological characteristics. These features attract more and more attention from various communities: scientific, medical, health, and industry. Success in the development of medicines to treat and potentially cure this cancer depends on active collaboration between these communities. It is anticipated that in the future genetic profiling will become a common and routine tool to design the most effective treatment and achieve the best therapeutic outcome for each individual patient. Given that the majority of patients present late in the disease and, until the very recent past, Stage IV lung cancer has carried an extremely limited and poor prognosis, the rapid progress made in recent years in the understanding and treating of ALK‐positive NSCLC brings promise and hope to all people affected by this condition. Finally, it is important to acknowledge the significance of social interactions by means of various media platforms and community groups. Such networks dedicated to ALK‐positive NSCLC patients have become an integral part of communications providing a confidential and supportive space to share personal experiences, offer advice, valuable resources and encouragement.

## Author Contributions

The author, Elena Klenova, is the sole contributor and was responsible for all aspect of this review: conceptualization, literature search and selection of relevant information, content organisation, illustrations and graphics design, writing – original draft preparation, review, revisions, editing, proofreading.

## Disclosure

This review (entitled “Molecular mechanisms and treatment strategies of ALK‐positive lung cancer: a beginner's guide for patients, their families and carers”) is based on an essay written by the author for a private patient group associated with the ALK‐positive Lung Cancer Charity (UK) in September 2022. It had not been previously peer reviewed or officially published. The ALK‐positive Lung Cancer Charity (UK) housed this article on their website between September 2022 and September 2025; this item has now been removed and will not be available and accessible. The current review is an expanded and updated version of this essay by the same author with no copyright infringement. The author takes sole responsibility for the content of the publication.

## Ethics Statement

The author has nothing to report.

## Conflicts of Interest

The author declares no conflicts of interest.

## Supporting information


**Data S1:** Supporting Information.

## Data Availability

Data sharing not applicable to this article as no datasets were generated or analyzed during the current study.
