# Supplementary material for: Molecular Mechanisms and Treatment Strategies of ALK‐Positive Lung Cancer: A Beginner's Guide for Patients, Their Families and Carers
Source: Thorac Cancer. 2025 Nov 10;16(21):e70182. doi: 10.1111/1759-7714.70182 (PMC12602093; doi:10.1111/1759-7714.70182)
Supplement: Supplementary file 1 — Data S1: Supporting Information. [file TCA-16-e70182-s001.docx]

**Molecular mechanisms and treatment strategies of ALK-positive lung cancer: a beginner’s guide for patients, their families and carers.**

**Elena Klenova**

**School of Life Sciences, University of Essex, UK: ALK Positive Lung Cancer Charity, UK; Oncogene Cancer Research Charity, UK.**

**Supporting Information**

# 13. Supplementary Figure 1 (S1). ALK Domain Structure and Regulatory Elements. The diagram depicts key features of the full length ALK protein (dimer on the left and monomer on the right). The extracellular domain comprises sections crucial for ligand binding and potential activation, including MAMs and LDLa domains. Within the intracellular protein kinase domain (PTK), essential regulatory segments governing ALK’s active and inactive states are highlighted, shedding light on its allosteric control and potential therapeutic targeting. Notably, the A-loop, housing pivotal amino acid residues Y1278, Y1282, and Y1283, drives ALK activation and downstream signaling. In contrast, specific C-terminal lysine residues serve as targets for methylation, contributing to regulatory functions. Furthermore, this figure delineates the duality of ALK functionality: ligand-dependent dimerization (Left) and ligand-independent monomeric activity (Right).

# Numbers correspond to amino acids in the ALK protein (1-1620).

# Figure is reprinted from Parvaresh et al [[1](#_ENREF_1)], *Copyright 2024, Published by MDPI.*


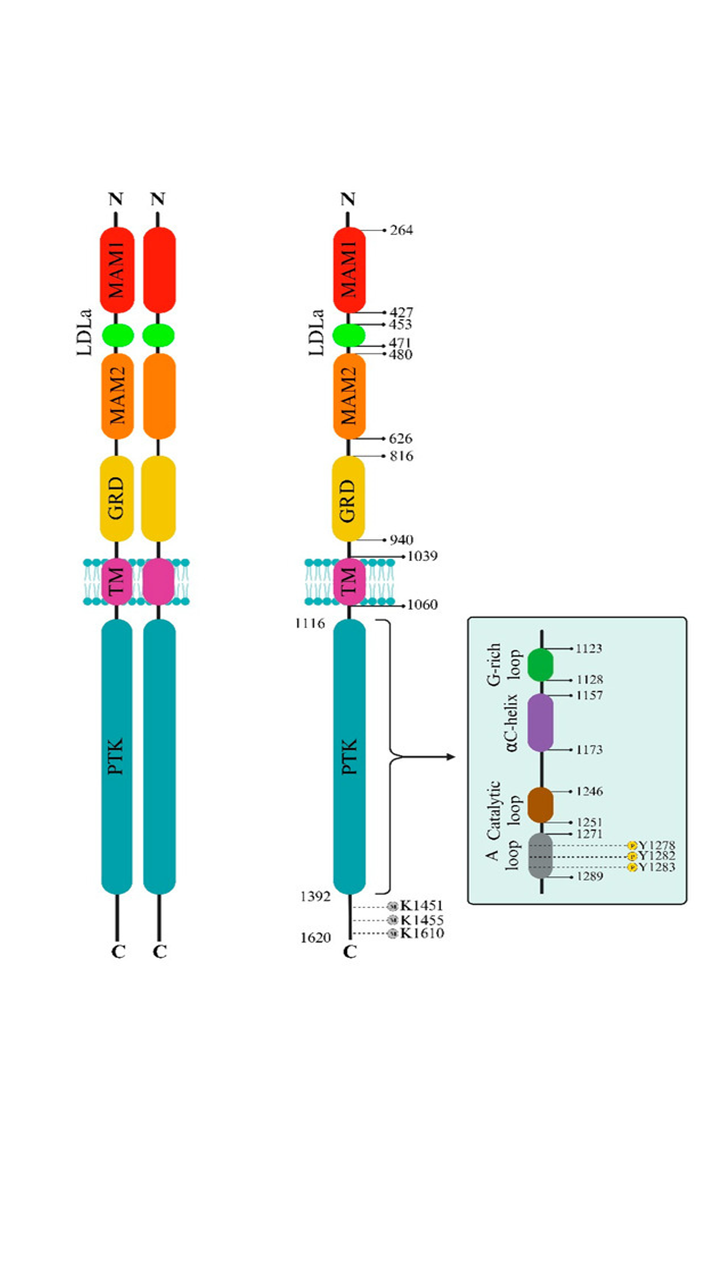


# 14. Supplementary Figure 2 (S2). Different variants of *EML4-ALK* fusion partners. Reprinted from Zhang *et al*, [[2](#_ENREF_2)], *Copyright 2021,Published by Elsevier B.V.*

1. Schema of the structural domains and fusion breakpoints of various “numbered” EML4-ALK variants. The nomenclature refers to the exon* in *EML4* translocated to the exon in *ALK*.
2. Distribution o EML4-ALK variants among EML4-ALK+ NSCLC pooled from two independent sequencing database.

The most common variants are E13;A20 (variant 1) and E6;A20 (variant 3a/b).

*Genes in the genome consist of exons and introns. Exons represent so called coding regions, which contain information for making a protein. Introns are non-coding regions, i.e. they do not contain information for making proteins.


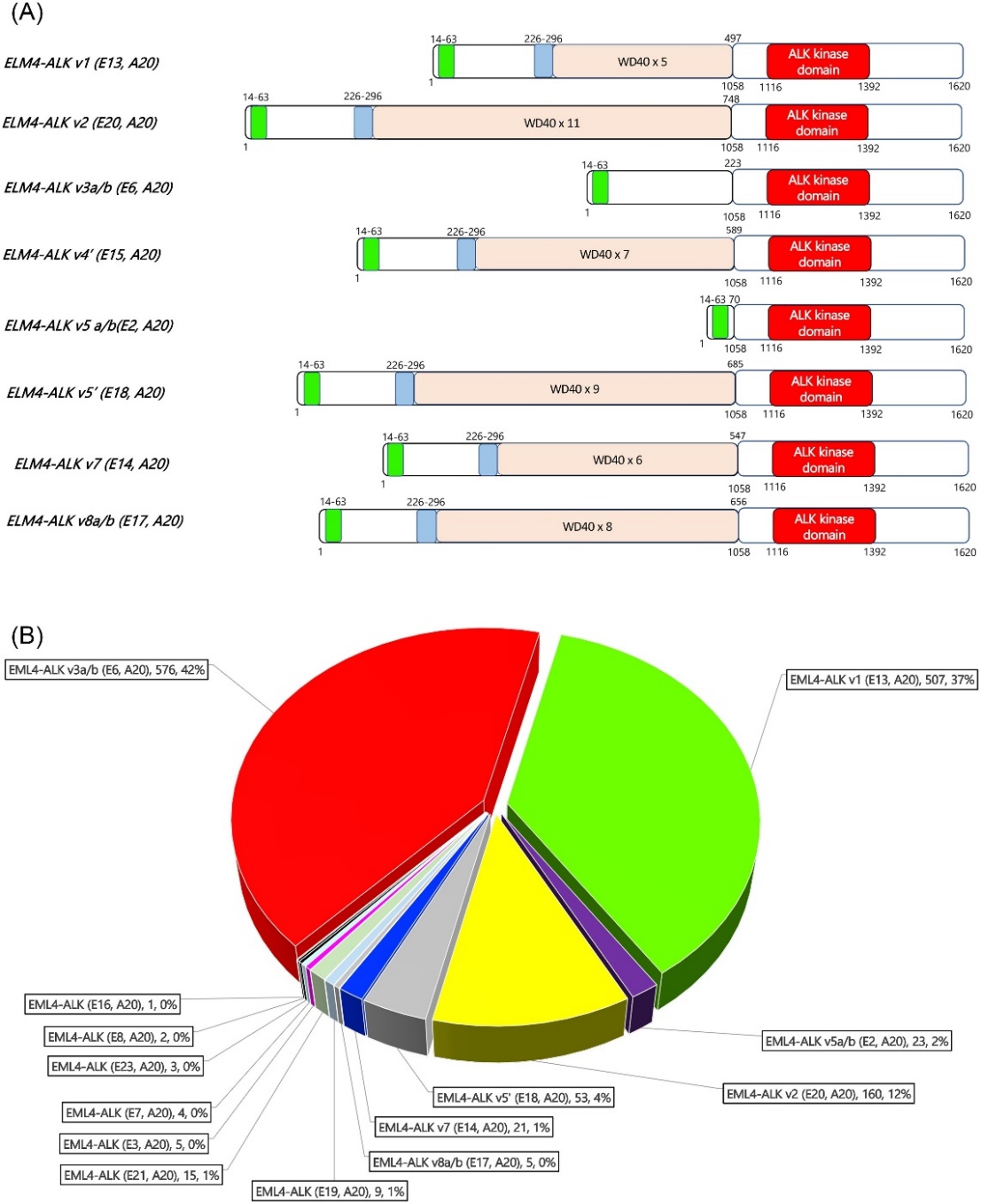


# 15 Supplementary Figure 3 (S3). Structures of different generations of ALK inhibitors. FDA approved ALK inhibitor drugs and novel macrocyclic ALK inhibitors. The structure and molecular weight (MW) of five FDA approved ALK inhibitor drugs (1st to 3rd generations) and newly developed macrocyclic ALK kinase inhibitors were presented. When there are no resistant mutations in ALK tyrosine kinase domain, ALK kinase activities can be effectively inhibited by all currently approved ALK tyrosine kinase inhibitor drugs (TKIs). The 2nd generation ALK TKI drugs can effectively inhibit the activity mutation L1196M, but they are not so effective on mutation G1202R and many compound mutations. The 3rd generation ALK TKI drug Lorlatinib is a small and compact macrocyclic inhibitor. It can effectively inhibit the activity of G1202R mutation but not for those compound mutations. Latest developed macrocyclic ALK TKI drugs (4^th^ generation) are even smaller and more compact than Lorlatinib. They are highly potent against G1202R mutation and many lorlatinib resistant compound mutations. (Adapted with modifications from Song et al [[3](#_ENREF_3)], *Copyright 2021 MedComm published by Sichuan International Medical Exchange & Promotion Association (SCIMEA) and John Wiley & Sons Australia, Ltd.*)

**Note that these inhibitors structurally mimic ATP** (graphic illustration and chemical diagram are shown in the box on top).

**ATP - graphic illustration**

**ATP - chemical diagram**


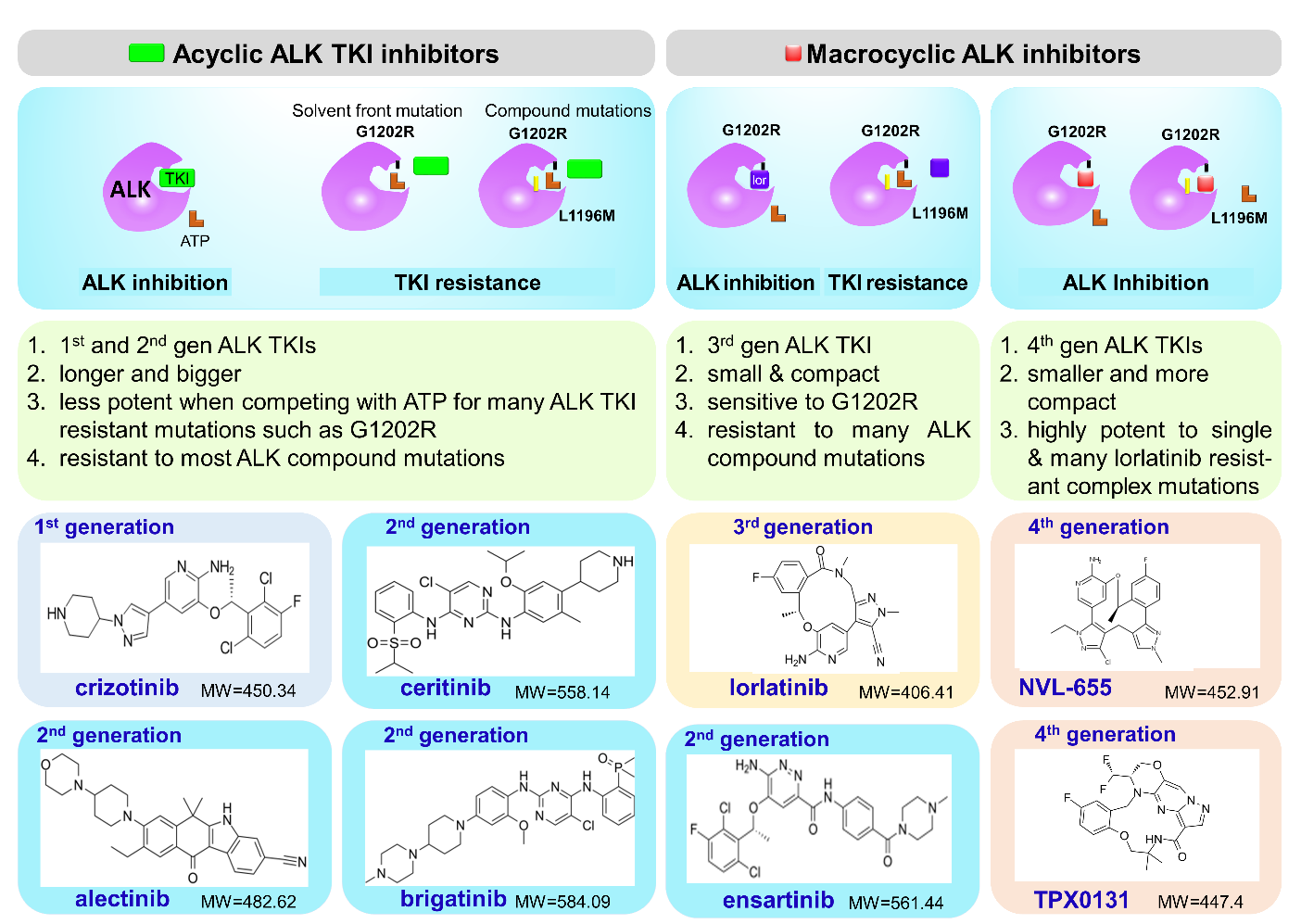


**16. Supplementary Figure 4 (S4). A typical example of ALK mutations identified by genetic testing. Distribution of ALK mutational resistance profiles according to the ALK inhibitor on which patients relapse**

Reused from Koopman et al [[4](#_ENREF_4)], *Copyright 2022 Published by Elsevier Inc.*

*(Note that the mutation identifier contains the information about the position of the mutation in the* ALK *protein and type of mutations. For example, G1269A means the change at the position 1269 in the protein from Glycine to Alanine. Note, that this position is within the TK domain (see the map of the* ALK *protein in* ***Supplementary Figure 1****).*


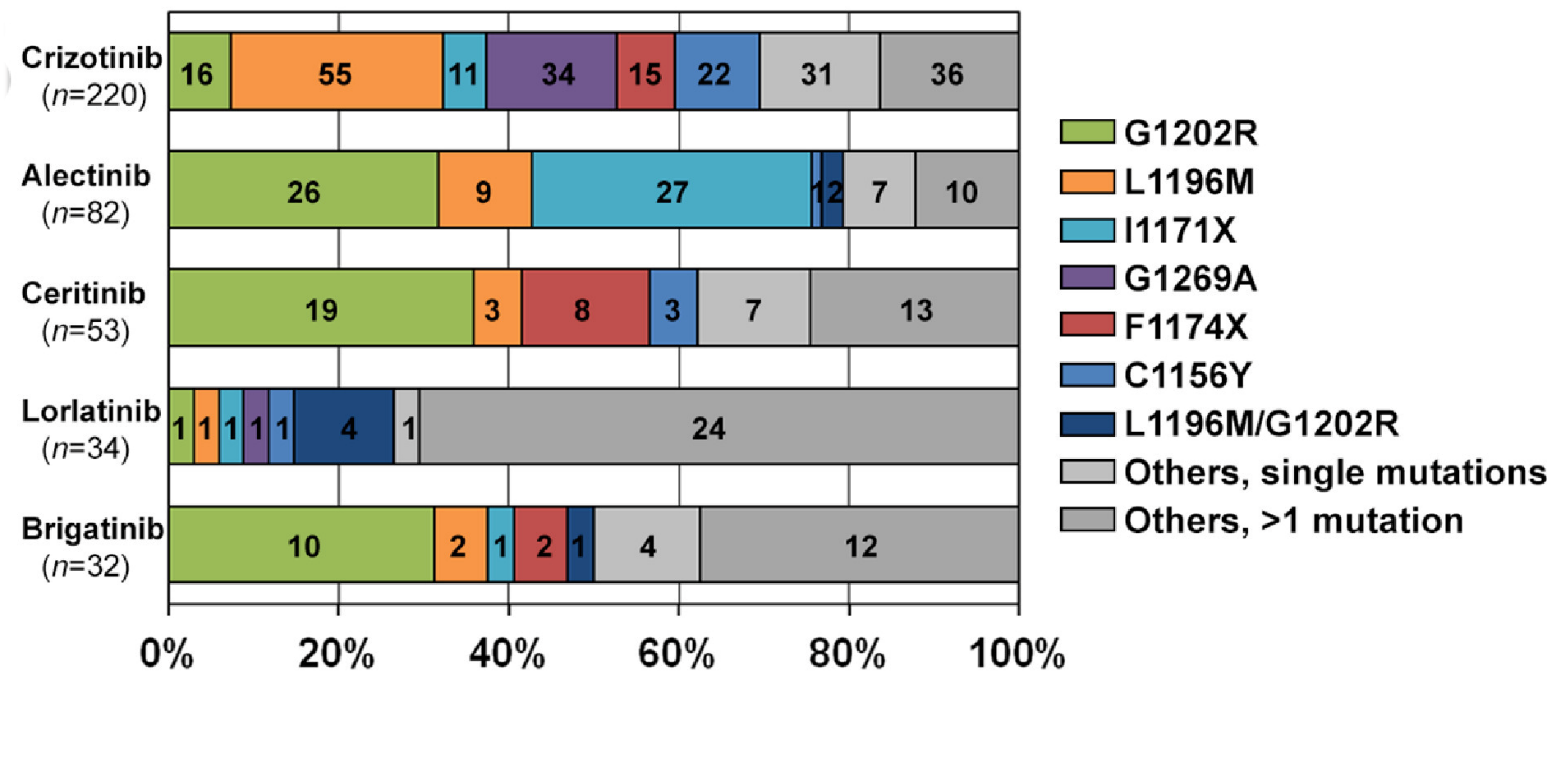


“Characteristics of the pooled population of 450 ALK inhibitor resistant, ALK fusion-positive NSCLC patients with acquired ALK mutations.

A marked difference was observed in the frequency of resistance mutations when stratifying according to ALK inhibitor administered prior to the biopsy.

**[Crizotinib-1G]** Resistance to first-line [crizotinib](https://www.sciencedirect.com/topics/medicine-and-dentistry/crizotinib) (n = 220) was associated with a wide range of individual mutations, with L1196M (n = 55 [25%]) and G1269A (n = 34 [15%]) occurring most frequently. These on-target mutations were observed more often with [crizotinib](https://www.sciencedirect.com/topics/pharmacology-toxicology-and-pharmaceutical-science/crizotinib) than with other inhibitors (P <.001 for both).

**[Alectinib- 2G]** Substitutions of I1171 (n = 27 [33%]) and G1202R (n = 26 [32%]) were the most common resistance-inducing mutations following treatment with [alectinib](https://www.sciencedirect.com/topics/pharmacology-toxicology-and-pharmaceutical-science/alectinib) (n = 82). I1171X was more common with alectinib than with other inhibitors (P <.001).

**[Ceritinib and Brigatinib-2G]** The on-target resistance mutations for [ceritinib](https://www.sciencedirect.com/topics/medicine-and-dentistry/ceritinib) (n = 53) and [brigatinib](https://www.sciencedirect.com/topics/medicine-and-dentistry/brigatinib) (n = 32) were comparable (P = .54), with G1202R as the most prevalent mutation (n = 19 [36%] and n = 10 [31%], respectively).

**[Lorlatinib-3G]** The third-generation ALK inhibitor [lorlatinib](https://www.sciencedirect.com/topics/medicine-and-dentistry/lorlatinib) (n = 34) had a very different spectrum of resistance-induced mutations, with the vast majority of samples (n = 24 [82%]) harboring more than one ALK mutation. The most common on-target resistance mechanism for lorlatinib was the L1196M/G1202R compound mutation (n = 4 [12%]).”

# 17. Supplementary Figure 5 (S5). Proposed treatment algorithm for ALK-rearranged advanced NSCLC.

Reprinted from Fukui et al, 2022 [[5](#_ENREF_5)], *Copyright 2022, Published by MDPI.*

#

“When the disease progresses during first-line therapy, the identification of resistance mechanisms by performing tissue/liquid biopsy may help to guide optimal treatment. For example, in the case that EML4-ALK G1202R is the cause of resistance, lorlatinib may be a favorable subsequent therapy, and the fourth-generation ALK-TKIs to be developed in the future are effective for compound ALK mutation. When an EGFR mutation is identified, combination therapy with ALK-TKI and EGFR-TKI may be effective, and crizotinib is a reasonable option for patients with confirmed MET amplification. If the disease has converted to small cell lung cancer, a different chemotherapy regimen is required from those for NSCLC.”

**
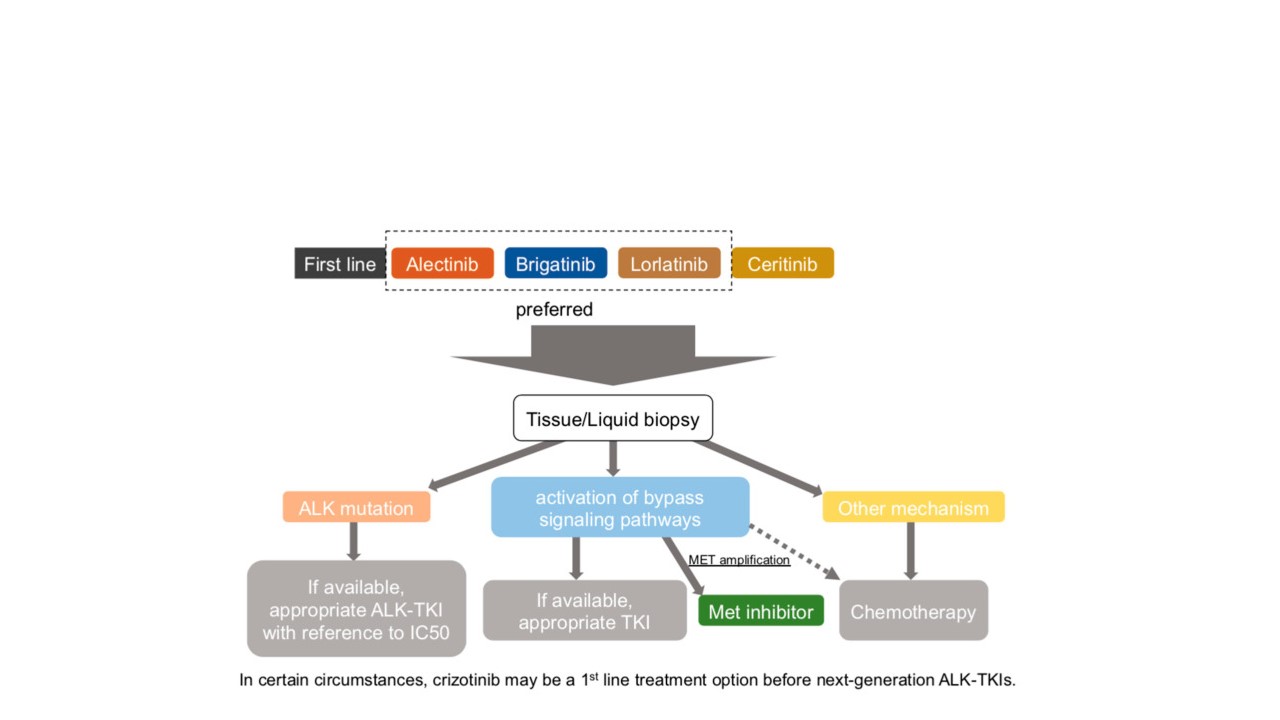
**

# References

1. Parvaresh H, Roozitalab G, Golandam F et al. Unraveling the Potential of ALK-Targeted Therapies in Non-Small Cell Lung Cancer: Comprehensive Insights and Future Directions. Biomedicines 2024; 12:

2. Zhang SS, Nagasaka M, Zhu VW et al. Going beneath the tip of the iceberg. Identifying and understanding EML4-ALK variants and TP53 mutations to optimize treatment of ALK fusion positive (ALK+) NSCLC. Lung cancer 2021; 158: 126-136

3. Song X, Zhong H, Qu X et al. Two novel strategies to overcome the resistance to ALK tyrosine kinase inhibitor drugs: Macrocyclic inhibitors and proteolysis-targeting chimeras. MedComm 2021; 2: 341-350

4. Koopman B, Groen HJM, Schuuring E et al. Actionability of on-target ALK Resistance Mutations in Patients With Non-Small Cell Lung Cancer: Local Experience and Review of the Literature. Clinical lung cancer 2022; 23: e104-e115

5. Fukui T, Tachihara M, Nagano T et al. Review of Therapeutic Strategies for Anaplastic Lymphoma Kinase-Rearranged Non-Small Cell Lung Cancer. Cancers 2022; 14:
